# Machine Learning-Based Analysis Reveals Triterpene Saponins and Their Aglycones in *Cimicifuga racemosa* as Critical Mediators of AMPK Activation

**DOI:** 10.3390/pharmaceutics16040511

**Published:** 2024-04-07

**Authors:** Jürgen Drewe, Verena Schöning, Ombeline Danton, Alexander Schenk, Georg Boonen

**Affiliations:** 1 Medical Department, Max Zeller Söhne AG, 8590 Romanshorn, Switzerland; ombelined@gmail.com (O.D.); alexander.schenk@zellerag.ch (A.S.); georg.boonen@zellerag.ch (G.B.); 2 Clinical Pharmacology and Toxicology, Department of General Internal Medicine, Inselspital—University Hospital, 3010 Bern, Switzerland

**Keywords:** AMPK activator, logistic regression classification, deep neural networks, machine learning, *Cimicifuga racemosa*, triterpene saponins, polyphenols

## Abstract

*Cimicifuga racemosa* (CR) extracts contain diverse constituents such as saponins. These saponins, which act as a defense against herbivores and pathogens also show promise in treating human conditions such as heart failure, pain, hypercholesterolemia, cancer, and inflammation. Some of these effects are mediated by activating AMP-dependent protein kinase (AMPK). Therefore, comprehensive screening for activating constituents in a CR extract is highly desirable. Employing machine learning (ML) techniques such as Deep Neural Networks (DNN), Logistic Regression Classification (LRC), and Random Forest Classification (RFC) with molecular fingerprint MACCS descriptors, 95 CR constituents were classified. Calibration involved 50 randomly chosen positive and negative controls. LRC achieved the highest overall test accuracy (90.2%), but DNN and RFC surpassed it in precision, sensitivity, specificity, and ROC AUC. All CR constituents were predicted as activators, except for three non-triterpene compounds. The validity of these classifications was supported by good calibration, with misclassifications ranging from 3% to 17% across the various models. High sensitivity (84.5–87.2%) and specificity (84.1–91.4%) suggest suitability for screening. The results demonstrate the potential of triterpene saponins and aglycones in activating AMP-dependent protein kinase (AMPK), providing the rationale for further clinical exploration of CR extracts in metabolic pathway-related conditions.

## 1. Introduction

Extracts of *Cimicifuga racemosa* L., NUTT. (also known as *Actaea racemosa* L. or black cohosh) are widely accepted [1,2,3,4] and have been granted “well-established use” status in the treatment of postmenopausal (i.e., climacteric) complaints by the European Medicines Agency [5]. This monograph predominantly includes vasomotor symptoms such as hot flushes and sweating, as well as nervousness, irritability, and metabolic changes. Although characteristic postmenopausal complaints have been known for a very long time and the beneficial effects of Cimicifuga extracts on climacteric symptoms are well accepted [3,4], the mechanism of actions has not yet been fully elucidated.

As well as clinical studies involving female patients, Seidlova-Wuttke et al. (2012) [6] undertook a comprehensive investigation aimed at delving into the beneficial impacts of a CR extract on postmenopausal symptoms in ovariectomized rats. In addition to the commonly reported climacteric effects, the authors were able to discern noteworthy reductions in fat accumulation and a decrease in the manifestations of metabolic syndrome in these animals. As AMP-activated protein kinase (AMPK) plays a pivotal role in regulating cellular metabolism [7], Moser et al. [8] investigated the effect of a CR extract Ze 450 and three of its isolated components (23-epi-26-deoxyactein, protopine, and Cimiracemoside C) on AMPK activity and carbohydrate metabolism in HepaRG cells and male *ob/ob* mice.

The extract and its components activated AMPK to the same extent as the AMPK activator metformin. The results also showed the extract led to significant reductions in body weight and plasma glucose levels, while improving glucose metabolism and insulin sensitivity in male diabetic *ob/ob* mice [8]. These findings broadened the mechanism of action of Cimicifuga in various domains to include the activation of AMPK and the subsequent effect on cellular metabolism, as indicated by a recent review discussion [9]. This new perspective brings new areas of application such as metabolic disorders, cardiovascular diseases, obesity, anti-aging, antioxidative, and supportive antiproliferative therapy into the focus of future clinical developments.

When examining the literature on published AMPK activators, the substantial chemical and pharmacological heterogeneity of the activators becomes evident. While only a handful of these (naturally occurring) activators directly target the enzyme itself, such as salicylate or AMP, the majority exert their effects indirectly. They achieve this by either influencing upstream kinases that subsequently phosphorylate AMPK or by reducing cellular ATP levels, leading to AMPK phosphorylation and subsequent activation. In particular, a variety of plant extracts or isolated plant constituents have been described in the literature to activate the enzyme [10,11,12].

The primary class of naturally occurring metabolites that may activate AMPK is the class of triterpene saponins and polyphenols such as flavonoids, courcumin, stilbenes, and others may also do so [13,14,15]. The class of triterpene saponins is widely distributed throughout the plant kingdom and constitutes a large and diverse group of secondary metabolites. They consist of a hydrophobic (water-repelling) aglycone, which can be steroidal or triterpenoid in nature, and one or more hydrophilic sugar moieties known as glycosides. These sugar moieties can be either monosaccharides or oligosaccharides and exhibit variations in their structure, size, and composition. The most common sugar moieties in steroidal saponins include glucose, galactose, rhamnose, xylose, and arabinose, which can undergo further metabolic processes. The type and number of sugar moieties attached to the steroid or triterpenoid aglycone affect the physicochemical properties and biological activities of the saponins, such as their solubility, stability, and bioavailability [16]. Saponins usually have unfavorable physiochemical properties for oral absorption due to their large molecular mass and hydrophilicity, which hinders enteral absorption and cellular uptake [17]. Hence, biotransformation to aglycones by cleavage of the glycosidic sugars may significantly alter cellular availability and consequently affect their pharmacological effects. Notably, certain saponins undergo deglycosidation by colonic microflora leading to enhanced intestinal absorption of the lipophilic aglycones. This is observed in the cases of certain ginsenosides and soybean saponins [18,19,20]. These compounds may also have a higher probability of entering their target cells.

When investigating herbal remedies, experiments can be challenging. The herbal extracts are complex and often contain multiple substances. Additionally, obtaining pure isolated compounds from these extracts can be difficult.

This presents an opportunity where machine learning models can significantly enhance the classification of activator constituents. Machine learning offers the possibility of thorough screening of these complex mixtures so that key compounds can be accurately identified, thereby streamlining subsequent detailed analysis and testing.

Recently, we have published research about sensitive and accurate machine learning models for the classification of AMPK activators [12]. In the present study, an extended and updated version of this applied database of known activators and controls has been used to classify all chemically characterized constituents of the Cimicifuga extract Ze 450 to estimate its ability to activate AMPK.

## 2. Materials and Methods

**Figure 1:**
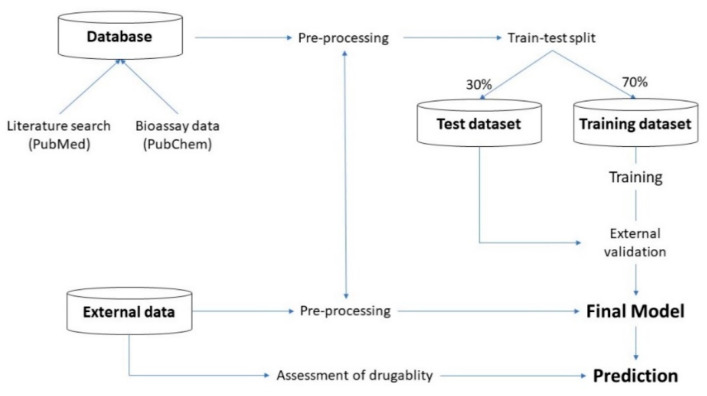
Flow and structure of experiments.

The flow and structure of experiments are illustrated in the following Figure 1:

### 2.1. Data

A highly detailed AMPK dataset was compiled in 2021 [13] and recently updated in August 2023. It was compiled by a thorough literature review of AMPK activators and inhibitors, conducted on PubMed (https://pubmed.ncbi.nlm.nih.gov/, accessed on 4 April 2024) using the search terms “AMPK AND activation” and “AMPK AND inhibition”. Compounds were included if they were confirmed activators or inhibitors by at least one publication listed on PubMed. Additionally, the Bioassay database of PubChem Substance and Compound databases (https://pubchem.ncbi.nlm.nih.gov/, accessed on 4 April 2024) was consulted, particularly when compounds exhibited an EC50 of ≤0.1 µM, indicating activation. Conversely, compounds that were tested and found to be inactive for AMPK activation or exhibited inhibitory activity were used as the control group for this analysis. In total, the database comprised N = 1120 and N = 815 active compounds or controls, respectively.

To comprehensively characterize the power of *Cimicifuga racemosa*, 95 chemically defined compounds from the rhizome were included for analysis [21] (see Table A1, Appendix B).

### 2.2. Data Preprocessing

Chemical structures were coded using the *simplified molecular-input line-entry system* (isomeric SMILES taken from PubChem). Data were used to calculate MACCS fingerprint descriptors (Molecular ACCess System, [22]). MACCS fingerprint descriptors are binary representations encoding the presence or absence of specific structural features or substructures within a molecule. They are represented by a fixed-length vector of 166 bits with “0” values indicating absence and “1” values indicating presence. They do not encode information about bond order, stereochemistry, or spatial arrangement of atoms. Despite these limitations, fingerprint descriptors are commonly used in cheminformatics and computational chemistry. Since MACCS fingerprints focus on specific structural features, they are effective at capturing chemical diversity in a dataset [23].

Finally, data preprocessing (curation) entailed eliminating duplicate entries, salts, mixtures, smaller fragments, and proteins from SMILES structures, with a focus on low molecular weight drug-like compounds (molecular weight < 1000). Lastly, tautomers were not standardized during this process.

To reduce computational effort and noise, the *VarianceThreshold feature selection* method was used to remove features with low variance (<0.01%).

The unbalanced distribution of activators and controls was compensated for by the *Synthetic Minority Oversampling Technique* (SMOTE, [24]), which generates synthetic samples for the minority class by interpolating between existing samples. It creates new samples that are combinations of neighboring samples, resulting in an even class distribution (1122 members for each class). SMOTE was only applied in the training and not in the test phase.

### 2.3. Validation

Validation of models was based on OECD Principles for (Q)SAR Validation [25] using the 2:1 random split of the 2244 total data into 1570 training and 674 test data. These training data were further split (5:1 ratio) into a validation training dataset (N = 1258) and a validation test dataset (N = 314) to optimize model hyperparameters and train the models (using the sklearn *train-test split* method). After completion of training, the test data served as an external control using 5-fold *cross-validation.* Furthermore, the training was repeated after randomization of the response variable (*Y-randomization* [26]).

The high-dimensional data of activators and controls were transformed into a two-dimensional space using the t-distributed stochastic neighbor embedding technique (tSNE). This method offers a visual representation of the structural relationship between various compounds, aiding in the interpretation of the database’s applicability domain [27].

### 2.4. Machine Learning Models

The following three machine learning techniques were applied: Deep Neural Networks, Logistic Regression Classification, and Random Forest Classification.

All calculations were performed using Python 3.11.2 (https://www.python.org/, accessed on 4 April 2024). Graphical analysis was carried out using OriginPro, version 2023, OriginLab Corporation, Northampton, MA, USA, or Matplotlib, version 3.3.3 (https://matplotlib.org/#, accessed on 5 April 2024).

#### 2.4.1. Deep Neural Network (DNN)

DNNs are sophisticated computational models with multiple interconnected layers, allowing them to automatically learn hierarchical representations of complex patterns from data [28]. Their depth enables effective feature extraction and is a key factor in their success across various machine learning tasks.

The data were assessed using a sequential DNN model, featuring a variable number of dense, hidden, and dropout layers, with HeNormal as the kernel initializer and Constant (value = 0) as the bias initializer. The activation functions employed were the exponential linear unit (ELU) for positive values and sigmoid for the output layers. Binary cross-entropy was utilized as the loss function. Details of the model are given in Appendix A.

#### 2.4.2. Logistic Regression Classification (LRC)

LRC [29] is a powerful and widely used statistical method for modeling the probability of a binary outcome based on one or more independent variables.

LRC is used to estimate the probability $\hat{p}$ that an instance belongs to a class:

(1) $$\hat{p}=h_{\theta }\left(x\right)=\sigma \left(\theta^{T}\cdot x\right),$$

using the logistic function:

(2) $$\sigma \left(t\right)=\frac{1}{1+e^{-t}}.$$

Binary classification for two classes denoted with 0 and 1 was obtained by

(3) $$\hat{y}=\sigma \left(t\right)=\left\{\begin{array}{c} 0,\;\;\hat{p}<0.5\\ 1,\;\;\hat{p}\geq 0.5 \end{array}\right.$$

The scikit-learn procedure was used (https://scikit-learn.org/stable/modules/generated/sklearn.linear_model.LogisticRegression.html, accessed on 4 April 2024).

#### 2.4.3. Random Forest Classification (RFC) 

RFC, an ensemble method (https://scikit-learn.org/stable/modules/generated/sklearn.ensemble.RandomForestClassifier.html, accessed on 4 April 2024), enhances generalizability and robustness by aggregating multiple base estimators, surpassing the performance of individual estimators such as decision trees. Each base estimator in the sequence aims to minimize the bias of the combined estimator. Renowned for classification tasks, RFCs are adept decision tree algorithms. Hyperparameters were optimized through grid search analysis, covering the number of estimators, maximum features utilized, maximum tree depth, minimum samples for split and leaf, and impurity criterion. Notably, no bootstrap sampling was employed in the process.

### 2.5. Hyperparameter Tuning

The hyperparameter tuning was performed on both the validation training dataset (N = 1258) and a validation test dataset (N = 314), which was derived with a 5:1 split using the *train-test split* method to optimize model hyperparameters and train the models.

Some of the adjustable hyperparameters of the investigated models were tuned by grid search, which was coupled with a 5-fold cross-validation (using sklearn *GridSearchCV* module), the others were kept in their default settings. Specifically for logistic regression, we focused on two key hyperparameters: the inverse of the regularization strength, denoted as “C”, and the penalty functions, which could be either “l1” (Lasso), “l2” (Ridge) regression, or “elasticnet” (a combination of “l1” (Lasso) and “l2” (Ridge)). These penalty functions help to control the impact of large coefficients in the model, thereby discouraging it from fitting noise into the data. Additionally, we determined the optimal solver among various options, which included the Newton-conjugate gradient optimization method (“*Newton-cg*”), the Limited-memory Broyden–Fletcher–Goldfarb–Shanno optimization method (“*lbfgs*”), a linear programming approach (“*liblinear*”).

For DNN, a grid search was performed on learning rate, batch size, number of hidden layers, and dropout layers.

### 2.6. Model Evaluation

The dataset underwent partitioning using the sklearn.model_selection preprocessing method *train_test_split*, allocating 30% for testing and 70% for training. Subsequently, a 5-fold cross-validation (CV) was performed.

To compare data distributions and assess the application domain, t-distributed stochastic neighbor embedding analysis was conducted via the sklearn.manifold.TSNE procedure. This technique transforms high-dimensional data into a 2-dimensional representation, facilitating graphical evaluation of applicability domains.

Machine learning model performance was evaluated using the following metrics:

Accuracy: (TP + TN)/(TP + TN + FP + FN);
Precision: TP/(TP + FP);
Sensitivity: TP/(TP + FN);
Specificity: TN/(TN + FP).

Here, TP represents true positives (correctly predicted activators), FP denotes false positives (incorrectly predicted activators), TN signifies true negatives (correctly predicted controls), and FN stands for false negatives (incorrectly predicted controls).

### 2.7. Prevention of Overfitting

Overfitting is a common problem in machine learning and statistical modeling, and it occurs when a model learns to perform very well on the training data but fails to generalize its predictions to new, unseen data.

One important risk factor is an unbalanced distribution of activators and controls in our database. This is an inherent problem in AMPK activation. Due to the importance of this activation, many potential activator compounds have been tested experimentally, whereas a much smaller number of negative controls (often inhibitors) have been investigated. This leads to a bias in the reported results within the literature. To significantly minimize the risk of overfitting, various methodical precautions were undertaken.

#### 2.7.1. Feature Selection

Since more complex models have a greater risk of model noise and are prone to overfitting, we simplified our models by eliminating those features that contribute information only marginally (e.g., have a variance threshold below 0.01).

#### 2.7.2. Cross-Validation

Cross-validation, especially the 5-fold variant during hyperparameter tuning followed by a 10-fold variant coupled to the ROC analysis (see below), is a machine learning technique that gauges predictive model performance and generalization. It does this by splitting the dataset into ten roughly equal parts or “folds”. The model is trained on nine of these parts and tested on the remaining one. This process is repeated ten times, with each fold serving as the test set once.

The performance metrics (in our case accuracy) from these ten rounds were then averaged to judge the model’s overall performance. It is a powerful method for comprehensively evaluating a model’s capabilities. It is more robust than a single train-test split because it examines how well the model generalizes different subsets of data.

#### 2.7.3. Regularization 

For logistic regression: an application of regularization techniques like *L1* (Lasso) or *L2* (Ridge) regression or *elastic net* option was used to penalize large coefficients in the model. This discourages the model from fitting noise into the data. The parameter *C* denotes the inverse of the regularization strength. The choice between these techniques was made in the tuning of hyperparameters by the grid search procedure. For DNN, dropout layers were evaluated.

#### 2.7.4. Early Stopping 

For DNN training, an early stopping procedure (*keras.callbacks* module *EarlyStopping,* https://www.tensorflow.org/api_docs/python/tf/keras/callbacks/EarlyStopping, accessed on 4 April 2024) was applied to monitor the training loss and halt training if there was no improvement for five consecutive epochs.

### 2.8. Receiver Operating Characteristic (ROC)

To assess the performance of a binary classifier regardless of thresholds, the *receiver operating characteristic* (ROC) curve and its corresponding area under the curve (AUC) scores were computed [30]. This evaluation was complemented with a 10-fold cross-validation to ensure the robustness and generalizability of the results.

### 2.9. y-Randomization

A final aspect of method validation is y-randomization. In this step, the DNN was applied to the molecular descriptors (denoted by X) unchanged, while the target y was randomized (null model). The performance was then measured. If the original model significantly outperformed the null model, it suggested a meaningful relationship between the molecular descriptors (X) and biological activity (denoted by y) in our dataset. In such a scenario, it provided confidence in the predictive power of our model. To enhance confidence further, this process was repeated 50 times.

### 2.10. Classification of Cimicifuga racemosa (CR) Constituents

Using the SMILES of the CR constituents, the same molecular descriptors were calculated for the database. While the database was fitted to a standardizer and transformed, the CR descriptors were only transformed using the same standardizer. Using the best-performing model of the training, the CR constituents were predicted as either AMPK activators or controls.

To calibrate these classifications, 50 randomly chosen samples of the positive and negative controls of the database were each also classified in the same run. The models were ranked by the number of misclassifications.

#### 2.10.1. Comparison of *Cimicifuga racemosa* (CR) Metabolites with Database

The best-performing model from the analysis was then employed to classify the transformed CR constituent descriptors. For each CR constituent, the five most similar members of the database were determined through pairwise calculation of cosine similarity scores (k) using scikit-learn (https://scikit-learn.org/stable/modules/generated/sklearn.metrics.pairwise.cosine_similarity.html, accessed on 4 April 2024):

$$k(x,v)=\frac{<x,y>}{\left\|x\right\|\cdot \left\|y\right\|},$$

where $\left\|\cdot \right\|$ denotes the Euclidian norm and <*x*,*y*> denotes the dot product of vectors *x* and *y*. It ranges from −1 to 1. Values of *k* > 0.8 were regarded as similar.

#### 2.10.2. Comparison of *Cimicifuga racemosa* (CR) Saponins with Their Estimated Aglycones

In total, 46 of the CR constituents were identified as saponins. Their original SMILES codes were theoretically deglycosylated, following the approach suggested by SwissADME [31], to generate new SMILES codes for their corresponding aglycones. These new SMILES codes were then used to generate descriptors from the estimated aglycones for classification.

#### 2.10.3. Assessment of Markers for Oral Absorption

A comparison between triterpene saponin constituents and their aglycones was conducted using the web tool SwissADME [31] available at http://www.swissadme.ch, accessed on 4 April 2024. This tool utilizes robust and predictive models for physicochemical properties, pharmacokinetics, and drug-likeness. It allowed us to estimate several parameters considered as indicators for the oral bioavailability of drugs, including molecular weight (MW), water solubility [32], topological polar surface area (TPSA; [33]), distribution coefficient XlogP [34], the number of violations of Lipinski’s rule of five [35], and the estimated lead-likeness [31].

## 3. Results

### 3.1. t-SNE Analysis

**Figure 2:**
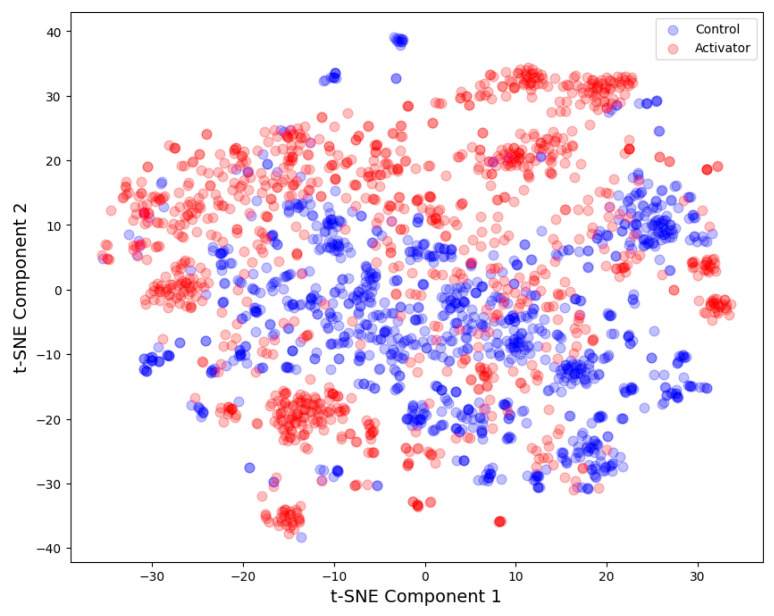
tSNE analysis: AMPK activators and controls. MACCS fingerprint descriptors (N = 2242, perplexity = 100, number of iterations = 5000).

**Figure 3:**
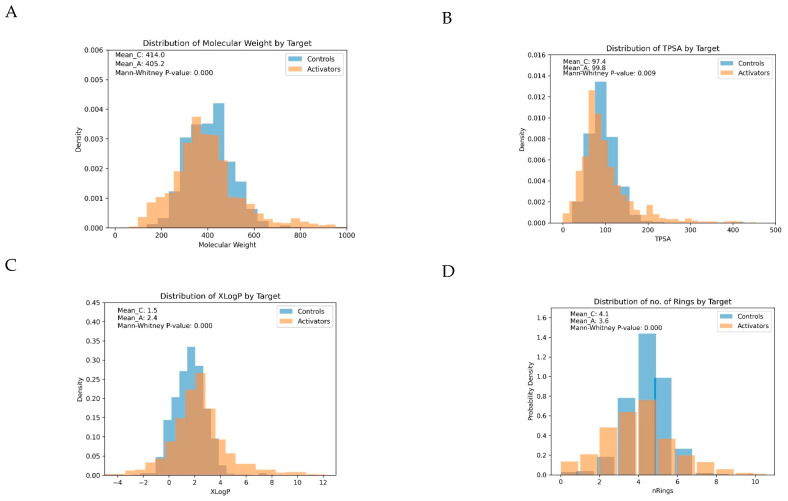
Distribution of four important physicochemical parameters between activators and controls: (**A**) Molecular Weight, (**B**) Total Polar Surface Area (TPSA), (**C**) Number of Rings in the Molecules, and (**D**) Predicted Octanol/Water Partition Coefficients (XLogP). Significant differences in the distributions of these parameters were observed (Mann–Whitney test): Activators had lower molecular weights (*p* < 0.0001), higher lipophilicity (median XLogP 2.4 for activators vs. 1.5 for controls; *p* < 0.0001), lower Total Polar Surface Area (TPSA) (*p* < 0.009), and fewer rings in the molecules, on average (3.6 for activators vs. 4.1 for controls; *p* < 0.0001), than controls.

The t-SNE graphical analysis indicates a clear separation between the two classes, namely activators and controls, across the MACCS fingerprint descriptors (Figure 2):

For illustration, the distribution of four important parameters between activators and controls is displayed in Figure 3:

### 3.2. Feature Reduction

Variance threshold reduction simplified the models by reducing the number of features to 139 for the MACCS fingerprint descriptors from their initial counts of 166.

### 3.3. Hyperparameter Tuning

For the MACCS fingerprint descriptors a batch size of 16, no dropout layers, a learning rate of 0.001, and three hidden layers were found to be optimal for the DNN model. As a solver, the *Adam optimizer* and the *binary cross-entropy* as loss functions were used.

For the LRC model, a regularization strength C of 0.5, a L2 penalty, and the liblinear solver were selected, and “newton-cg” for the solver was estimated to be optimal parameters. For RFC, the gini criterion was chosen, the maximum features were set to log2 (number of features), the min_samples_leaf and min_samples_split were set to 1 and 4, respectively, and the number of estimators was set to 110.

All other parameters were left at their default settings.

### 3.4. Test Performances

In evaluating the performance of various machine learning techniques, all models demonstrated a commendable accuracy level of approximately 90%. Notably, the DNN model exhibited superior performance compared with other models by minimizing the number of misclassifications on the calibration data. With DNN, there were only three misclassifications, in contrast to 17 for LRC and 9 for the RFC model.

While the LRC model achieved the highest overall test accuracy at 90.2%, both the DNN and RFC models surpassed it in terms of precision, sensitivity, specificity, and ROC AUC, as summarized in Table 1.

All models utilized the MinMax Scaler for data scaling prior to modeling. As a side note, the RFC model was also evaluated without prior scaling, producing identical results to those obtained with scaled data.

**Figure 4:**
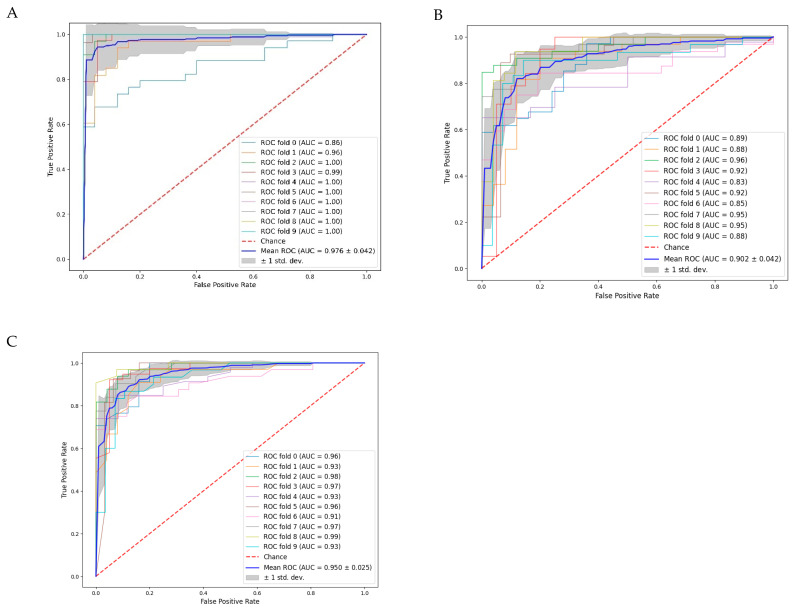
(ROC) analysis coupled with 10-fold cross-validation: (**A**) Deep Neural Network (DNN); (**B**) Logistic Regression Classification (LRC); and (**C**) Random Forest Classification (RFC).

The area under the receiver operating characteristic curve (ROC AUC) assesses a model’s capacity to differentiate between activator and control classes across various thresholds. These curves (Figure 4) were combined using a 10-fold cross-validation. A higher ROC AUC value indicates better class discrimination, with the optimal value being 1.0 or −1.0.

### 3.5. y-Randomization

Notably, in none of the 50 shuffled models could a distinction be made between activators and controls (see Table 1). The mean accuracy ranged from 57.6% ± 1.8% to 57.8% ± 1.8%. These results suggest that the unchanged models are statistically significant and are unlikely to have arisen by chance. This provides confidence in the predictive power of our models.

### 3.6. Classification of Cimicifuga racemosa (CR) Constituents

For classification, 103 chemically defined CR root compounds were identified [21] and checked for isomeric SMILES codes by using the PubChem database (https://pubchem.ncbi.nlm.nih.gov/, accessed on 4 April 2024). In total, 95 distinct compounds with all information available were used for analysis (see Table A1, Appendix B).

All compounds with triterpene and triterpenoid structures were classified as active. This classification is supported by the literature for 23-*Epi*-26-deoxyactein and cimiracemoside C [8]. From the non-triterpene compounds, the cinnamic, benzoic, or fukiic acid derivatives were clearly classified as active. A literature search supported this classification for synaptic acid [36], P-coumaric acid [11], isoferulic acid [37], protocatechuic acid [37], and protocatechuic aldehyde [38]. Compounds such as cimiracemates, cimiphenones, cimifugic acid derivatives, and actealactone were likewise classified as active. Among the chromones—angelicain, cimifugin, and visnagin—only angelicain and cimifugin were classified as active, whereas visnagin was classified as inactive, possibly due to the absence of a propan-2-ol group. Interestingly, the glycoside cimidahurin was classified as active. However, its aglycone hydroxytyrosol, and not the compound itself, was identified in the literature as an activator of AMPK [39]. For the chemical structures, see Appendix B: Table A1.

**Figure 5:**
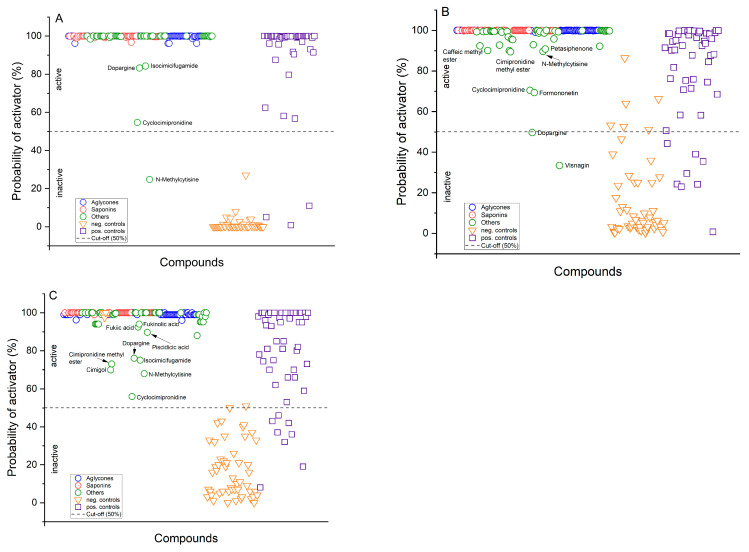
Classification of *Cimicifuga racemosa* root constituents was performed using three different methods: (**A**) Deep Neural Network (DNN); (**B**) Logistic Regression Classification (LRC); and (**C**) Random Forest Classification (RFC). Saponins and their aglycones are clearly classified as activators of AMPK. Saponins and their aglycones were unequivocally identified as activators of AMPK. While other constituents were also categorized similarly, albeit with lower probabilities. Among these constituents, cyclocimipronidine and dopargine were classified with uncertainty, along with N-methylcytisine, which the DNN model classified as inactive.

Further support for these classifications came from a similarity comparison of the CR constituents against our database. The constituents demonstrated high similarity to database compounds, with median similarity scores descending from 0.94 to 0.91. However, five compounds—cimipromidine (0.78), cimipromidine methyl ester (0.74), dopargine (0.77), and N-methylcytisine (0.797)—recorded the lowest similarity scores, aligning with their lower probability estimates of AMPK activation, as indicated in Figure 5. These findings, including individual similarity scores, are detailed in Table A2 in Appendix B, underscoring the data supporting the classification outcomes.

### 3.7. Comparison of Saponins with Their Aglycones

The 46 theoretical aglycones showed no systematic and significant differences in their probability compared with the saponins from which they were derived [31].

**Figure 6:**
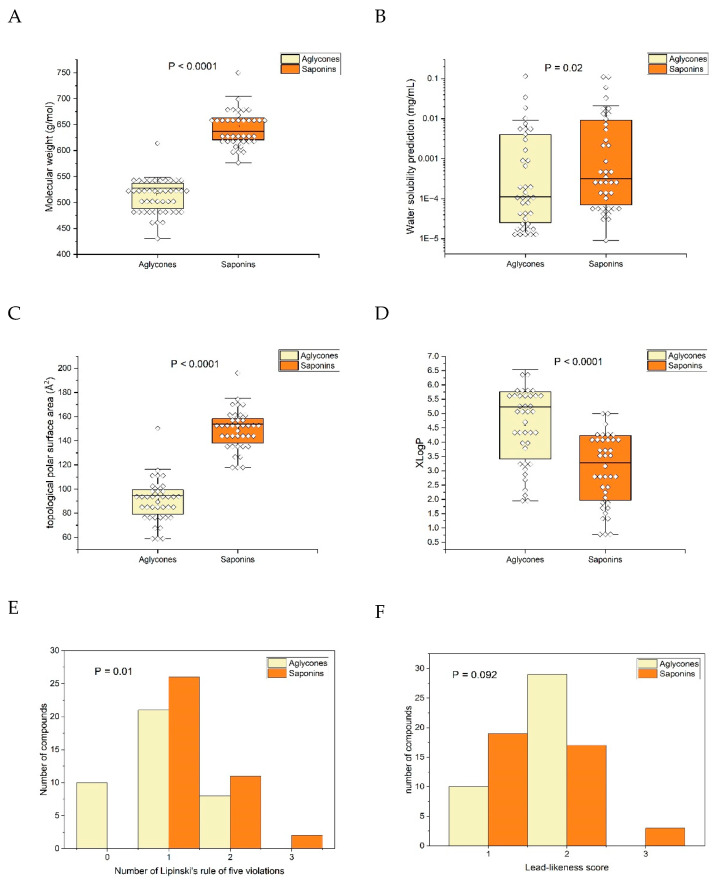
Comparison of triterpene saponin constituents with their theoretically derived aglycones (applied from open source SwissAMDE Webtool, [31]): (**A**) molecular weight of aglycones was significantly smaller than that of saponins (*p* < 0.0001, paired two-sided *t*-test); (**B**) water solubility surprisingly showed high overlap but was significantly smaller (*p* = 0.02); (**C**) topological polar surface area (TPSA) was clearly significantly smaller in the aglycones (*p* < 0.0001; paired two-sided *t*-test); (**D**) lipophilicity, as expressed by XLogP, increased significantly (*p* < 0.0001; paired two-sided *t*-test); (**E**) Lipinski’s rule of five violations was significantly differently distributed (*p* = 0.01; Wilcoxon signed-rank test), with aglycones having a smaller number of violations; and (**F**) estimation of the lead-likeness score was not significantly different.

Saponins and their corresponding aglycones were analyzed for several markers indicative of oral bioavailabilities and drug-likeness (Figure 6). Data were applied to open source Webtool SwissADME [31], available at http://www.swissadme.ch, accessed on 4 April 2014.

As constructed, the molecular weight of aglycones was consistently lower than their corresponding saponins. While water solubility exhibited a significant decrease, on average (*p* = 0.02, paired two-sided *t*-test), compared with the solubility of saponins, there was a notable overlap between the two groups. In contrast, the topological polar surface area showed minimal overlap and a highly significant difference (*p* < 0.0001, paired two-sided *t*-test) between aglycones and saponins. An increase in lipophilicity, as indicated by the significant elevation of XLogP (*p* < 0.0001, paired two-sided *t*-test), was evident.

Assessing oral bioavailability using Lipinski’s rule of five [35], which indicates improved bioavailability if all five conditions are met, revealed significantly fewer violations for the aglycones (*p* = 0.01, Wilcoxon signed-rank test). Despite expectations that the observed effects on topological polar surface area (TPSA) and XLogP would manifest as clear differences in water solubility, the substantial overlap in solubility suggests that various physicochemical parameters exert opposing effects. This phenomenon cannot be solely explained by lipophilicity in a monocausal manner. Concerning drugability (lead-likeness), no clear advantage of the aglycones over the saponins could be demonstrated (*p* = 0.09, Wilcoxon signed-rank test).

## 4. Discussion

Herbal preparations encompass complex mixtures of potentially active chemical compounds. Nevertheless, comprehensive in vitro experiments often necessitate pure, isolated substances for each identified constituent. Regrettably, such isolated constituents are frequently insufficiently available. Hence, our extended approach uses machine learning tools, offering novel opportunities to screen these multi-substance preparations and identify promising lead compounds. These can then undergo rigorous subsequent testing.

Even when availability problems are set to one side, directly assessing each ingredient in vitro is a resource-intensive and time-consuming endeavor. A swifter, more cost-effective solution could be employing diverse machine learning models. These models, based on an established structure–activity database, can predict the AMPK activation potential of numerous so far uncharacterized substances “in a single run”.

All models investigated showed very good performance in discriminating AMPK activators from controls. Surprisingly, with the exception of three compounds (cyclocimipronidine, dopargine, and N-methylcytisine), all of the 95 investigated CR constituents were clearly predicted activators. It was therefore necessary to rule out a technical artifact caused by the overfitting of the model. Overfitting is a common problem in machine learning and statistical modeling, and it occurs when a model learns to perform very well on the training data but fails to generalize its predictions to new, yet unseen data. In other words, an overfitted model has focused on capturing the noise or random fluctuations in the training data instead of accurately capturing the underlying patterns or relationships.

A risk factor for overfitting is an unbalanced distribution of activators and controls in our database. This is an inherent problem in pharmacology. Due to the importance of AMPK activation, many potential activator compounds have been experimentally tested, whereas a much smaller number of negative controls (often inhibitors) have been investigated. This leads to a bias in the reported results within the literature.

In mitigating the challenge of overfitting, various methodological measures have been implemented to minimize this risk:

1. Balancing unevenly distributed dataset classes;
2. Employing simpler models;
3. Implementing cross-validation;
4. Utilizing regularization techniques;
5. Employing early stopping techniques.

All of these precautions were rigorously applied to ensure that technical and methodological safeguards had been implemented.

As we have previously demonstrated [12], the positive controls within our dataset, which serve as activators, exhibit a notable structural diversity. This diversity arises from the fact that a significant proportion of activators exert their effects indirectly. They interact with regulatory sites upstream in the biological pathways. When these sites are activated, they, in turn, trigger the phosphorylation and activation of AMP-activated protein kinase (AMPK). AMPK is a critical enzyme responsible for sensing and regulating energy supply, as well as various cellular functions. These functions include controlling carbohydrate entry and metabolism, generating reactive oxygen species (ROS), regulating apoptosis, modulating cellular growth, and influencing processes like mitochondrial biogenesis and autophagy.

While we achieved an excellent predictive performance on our unseen test dataset, it is important to acknowledge that the presence of unaccounted-for mechanisms cannot be ruled out. It is also worth noting that machine learning models have inherent limitations. They provide classification *probabilities* that ideally should be validated through direct in vitro or in vivo experiments or by other evidence. Another limitation is the research process itself. It focuses on AMPK activators rather than inhibitors or inactive substances. As a result, significantly fewer substances have been identified that inhibit AMPK, or, perhaps even more importantly, are confirmed not to interact with it. This leads to a selection bias in our database and unbalanced distribution and thus poses a theoretical risk of over-identifying active substances. This suggests that external evidence should also be sought.

A point that clearly supports the validity of the classifications is the calibration of the data, each consisting of 50 randomly selected positive and negative controls. Their classifications were clearly separated, with only 3% to 17% misclassifications across the three models under investigation. Another point to consider is the high sensitivity (84.5–87.2%) and specificity (84.1–91.4%), which provide strong indications for suitability as a screening tool.

To further substantiate our model’s predictive accuracy regarding the classification of the 95 CR constituents as either activators or controls, a comprehensive similarity analysis against all compounds in our database was performed. This involved computing the structural similarities of the CR constituents to every database entry and identifying the five most closely matching compounds for each metabolite (details provided in Table A2 in Appendix B). Notably, each of the CR constituents displayed considerable structural similarity to the positive control compounds within our database. The constituents showed high similarity to compounds in the database, with median similarity scores ranging from 0.94 down to 0.91. Nonetheless, a subset of compounds—specifically, cimipromidine (0.78), cimipromidine methyl ester (0.74), dopargine (0.77), and N-methylcytisine (0.797)—registered the lowest similarity scores. This correlates with their diminished likelihood of activating AMPK, as reflected in the probability estimates presented in Figure 4. These observations, including individual similarity scores, are thoroughly documented in Table A2 in Appendix B, providing a robust data foundation supporting our classification results.

Studying herbal drugs presents a unique set of challenges due to the complexity of herbal extracts, which consist of multiple substances. Additionally, obtaining pure substances from these extracts is often a challenging task, resulting in limited availability. Consequently, our improved method offers exciting new prospects for conducting thorough analyses of these complex mixtures. It enables the examination of multi-component herbal extracts to identify particular compounds of interest. Subsequently, these compounds can undergo more extensive assessments and evaluations, followed by further refinement of the extracts to enhance the concentration of the desired components.

Our results indicate that the models clearly classified all constituents of *Cimicifuga racemosa* as activators apart from three non-triterpenes. This suggests a high probability of their ability to activate AMPK. However, we cannot determine the strength of this activation from our findings. Moreover, it is plausible that this activation is a collaborative or even synergistic effect, considering that many constituents were classified as active. The overall effect is certainly influenced by the concentrations of these active compounds at the site of action, which is hard to predict.

It is perplexing that the models made no distinction between triterpene saponins and their aglycones in terms of the probability of classifying the compounds as activators. Although it is conceivable that aglycones, due to their higher lipophilicity, have a greater likelihood of being absorbed into tissues and reaching the site of action [40], our model merely predicts whether the compounds are capable of activating AMPK at all. It does not take into account the dose–response relationship and kinetics.

Triterpene saponins, known for their high hydrophilicity, exhibit limited oral absorption from the gastrointestinal tract, especially when compared to their respective lipophilic aglycones (for a review, see [40]). In our experiments, the range of water solubility values of CR triterpene saponins significantly overlapped the range of the values of their corresponding aglycones, suggesting that this statement likely needs to be assessed individually for each saponin and aglycone. Consequently, it is difficult to predict the overall oral absorption of a multicomponent mixture as an herbal extract.

In current *Cimicifuga racemosa* extracts, the aglycone content is relatively low. Nevertheless, research has demonstrated that a significant portion of the dose of triterpene saponin, as observed with 23-epi-26 dihydroxyactein, is orally absorbed in both rats [41] and humans [42]. Nonetheless, following oral administration, certain triterpene saponins have the potential to reach the large intestine, where they might undergo degradation by the colonic microbiome. This process, similar to what has been observed for other triterpene saponins [40]), could also contribute to the overall effect.

This study has some limitations: While MACCS (Molecular Access System) descriptors are widely utilized in cheminformatics and machine learning for representing chemical compounds [23], it is essential to acknowledge their inherent limitations and potential biases. Being rooted in predefined substructures, there is a possibility of bias towards specific compound types or functional groups, potentially overlooking less common or innovative structural motifs. The reliance on a fixed set of molecular features may impede the generalizability of machine learning models across diverse chemical datasets. Furthermore, some MACCS descriptors may exhibit high correlation or redundancy, leading to multicollinearity in the feature space. Addressing such issues is crucial as it can impact the stability and interpretability of machine learning models, often necessitating feature selection or dimensionality reduction techniques, as we applied in our study.

Moreover, MACCS descriptors are primarily tailored for small organic molecules and may not adequately represent complex biomolecules or materials. Hence, to ensure compatibility with the descriptor’s scope, we constrained our dataset to small compounds (molecular weight ≤ 1000).

A PubMed search using the terms “AMPK” and “QSAR” reveals that various QSAR models for predicting AMPK activation have been documented [43,44]. These models predominantly rely on pharmacophore docking, homology modeling, and structure-, ligand-, or fragment-based design strategies, focusing solely on compounds that activate AMPK directly. Diverging from these methodologies, our research appears to be the first to comprehensively incorporate compounds that activate AMPK, regardless of whether the activation is direct or indirect. This inclusive approach enables a broader understanding and captures the diverse mechanisms of AMPK activation more effectively, addressing the enzyme’s activation heterogeneity.

## 5. Conclusions

The results of this study confirm that all triterpene saponins, as well as their aglycones, tested may contribute to activating the AMP-dependent protein kinase (AMPK). With regard to the mechanism, this may suggest a collaborative or even synergistic action on the enzyme. Since AMPK plays a pivotal role in various interconnected metabolic pathways, our results further underscore the rationale for clinically investigating the therapeutic benefits of *Cimicifuga racemosa* extracts in conditions associated with disturbances in these metabolic pathways.

## Figures and Tables

**Table 1:** Summary of results of classification of different machine learning methods.

Method	Training Accuracy (%)	Test Accuracy (%)	Y-Randomization (**)	Precision (%)	Sensitivity (%)	Specificity (%)	ROC AUC (*)	TN	FN	FP	TP
Deep Neural Network (DNN)	96.9	86.2	57.6 ± 1.8	89.8	86.0	86.5	97.6 ± 4.2	50	3	0	47
Logistic Regression Classification (LRC)	90.2	90.2	57.7 ± 1.5	87.9	84.5	84.1	90.2 ± 4.2	43	10	7	40
Random Forest Classification (RFC)	99.7	89.0	57.8 ± 1.8	93.3	87.2	91.4	95.0 ± 2.5	49	8	1	42

Dataset (number): activators (1120), controls (815, after SMOTE oversampling 1122). (*) ROC AUC = area under the receiver operating characteristics curve. (**) N = 50 permutations, TN = number of correctly classified controls, FN = number of falsely classified positive controls, FP = number of falsely classified negative controls, and TP = number of correctly classified positive controls.

## Data Availability

A complete list of used activators and controls is given in Supplementary Materials as Tables S1–S3, and source codes of all models are given in Table A1 and Table A2.

## Supplementary Materials

The following supporting information can be downloaded at: https://www.mdpi.com/article/10.3390/pharmaceutics16040511/s1; Table S1: DB_descript_MACCS; Table S2: Cimi_descript_MACCS; Table S3. Experiments.

## Appendix A. Details of the Deep Neural Networks Model

Details of the Deep Neural Networks model;
Python code: Model.ipynb;
Database: database.csv.

from sklearn.model_selection import KFold
from sklearn.metrics import make_scorer, accuracy_score
from keras.models import Sequential
from keras.callbacks import ModelCheckpoint
from keras.models import load_model
from sklearn.model_selection import GridSearchCV
from sklearn.model_selection import cross_val_score
n_features = X_train.shape [1]
n_targets = 1
learning_rate = 0.01
n_hidden = 4
batch_size = 32
epochs = 10
def create_model (n_features: int, learning_rate: float, n_hidden: int, batch_size: int,
dropout: float) -> Sequential:inputs = Input (shape = (number of features))
x = Dense (1_500, kernel_initializer = init_w, bias_initializer = init_b) (inputs)
x = Activation (“elu”)(x)
x = Dropout (dropout)(x)
for i in range (0,n_hidden):
(x) = Dense (1_500-i*300, kernel_initializer = init_w, bias_initializer = init_b) (x)
(x) = Activation (“elu”) (x)
(x) = Dropout (dropout) (x)
outputs = Dense (n_targets, activation = “sigmoid”) (x)
model = Model (inputs = inputs, outputs = outputs)
model.compile (loss = ‘binary_crossentropy’, optimizer = Adam(learning_rate = learning_rate), metrics = [‘accuracy’])
return model

## Appendix B

**Table A1:** Major constituents of *Cimicifuga racemosa* extracts.

Shengmanol type (16-ketone type)								
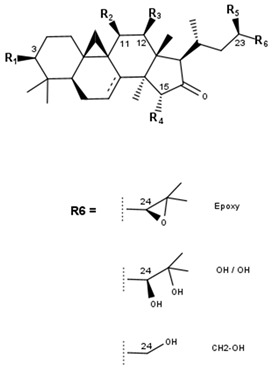
Compounds	R1	R2	R3	R4	R5	R6	Δ^7,8^	CID
23-*O*-Acetylshengmanol	OH	H	H	OH	O-Ac	Epoxy	-	91827092
23-*O*-Acetylshengmanol-3- *O*-β-*D*-xylopyranoside	O-Xyl	H	H	OH	O-Ac	Epoxy	-	56962372
23-*O*-Acetylshengmanol-3-*O*-α-L- arabinopyranoside	O-Ara	H	H	OH	O-Ac	Epoxy	-	10865257
Bugbanoside C	O-Ara	H	O-Ac	OH	=O	OH/OH	+	15894670
Bugbanoside D	O-Ara	H	O-Ac	OH	=O	Epoxy	+	15894671
Bugbanoside E	O-Ara	H	O-Ac	H	=O	Epoxy	+	15894672
Cimicifugoside H-1	O-Xyl	OH	H	H	=O	Epoxy	+	15241163
Cimicifugoside H-3	O-Xyl	OH	H	H	=O	CH_2_OH	+	15241164
Cimiracemoside L	4′-O-Ac-Xyl	H	H	OH	O-Ac	Epoxy	-	10952624
Cimicidanol	OH	OH	H	H	=O	Epoxy	+	10413064

Hydroxyshengmanol type									
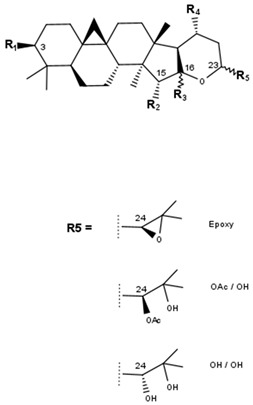
Compounds	R1	R2	R3	R4	R5	16	23	24	CID
24-Acetylhydroshengmanol-3-*O*-β-D- xylopyranoside	O-Xyl	OH	OH	CH_3_	O-Ac/OH	S	S	S	157168
Cimiracemoside E	O-Xyl	=O	H	CH_2_OH	O-Ac/OH	R	R	S	91827210
Shengmanol	OH	OH	OH	CH_3_	Epoxy	S	R	S	101133349
Shengmanol-3-*O*-β-D-xylopyranoside	O-Xyl	OH	OH	CH_3_	Epoxy	S	R	S	158275

Cimigenol type (A)								
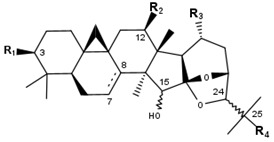
Compounds	R1	R2	R3	R4	Δ^7,8^	15	24	CID
Cimigenol	OH	H	CH_3_	OH	-	R	S	16020000
Cimigol	OH	H	CH_3_	OH	-	S	R	101596828
25-*O*-Acetylcimigenol	OH	H	CH_3_	O-Ac	-	R	S	46881255
25-*O*-Acetylcimigenol 3-*O*-α-L- arabinopyranoside	O-Xyl	H	CH_3_	O-Ac	-	R	S	24721386
25-*O*-Methylcimigenol	OH	H	CH_3_	O-CH_3_	-	R	S	146027510
25-*O*-Methylcimigenol-3-*O*-β-D- xylopyranoside	O-Xyl	H	CH_3_	O-CH_3_	-	R	S	146027510
25-*O*-Ethylcimigenol-3-*O*-β-D- xylopyranoside	O-Xyl	H	CH_3_	O-CH_2_CH_3_	-	R	S	16091662
12-β-Acetoxycimigenol	OH	O-Ac	CH_3_	OH	-	R	S	16104912
12-β-Acetylcimigenol-3-*O*-β-D- xylopyranoside	O-Xyl	O-Ac	CH_3_	OH	-	R	S	44418831
12-β-Hydroxycimigenol	OH	OH	CH_3_	OH	-	R	S	10006332
Bugbanoside F	O-Ara	OH	CH_3_	OH	+	R	S	101096469
Cimiracemoside B	O-Xyl	H	CH_2_OH	OH	-	R	S	91826883
Cimiracemoside C (=Cimifugoside M)	O-Ara	H	CH_3_	OH	-	R	S	15541911
Cimiracemoside D	O-Ara	O-Ac	CH_3_	OH	-	R	S	70698290
Cimiside A	O-Xyl	OH	CH_3_	OH	-	R	S	91827183
Cimiside B	3′-O-Xyl-3-O-Xyl	H	CH_3_	OH	-	R	S	10054869

Cimigenol type (B)				
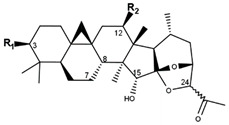
Compounds	R1	R2	24	CID
25-AnhydroCimigenol-3-O-α-L- arabinopyranoside	O-Ara	H	R	70698285
Cimiracemoside J	O-Ara	O-Ac	S	10952455
Cimiracemoside K	O-Xyl	O-Ac	S	10930352
Cimiside E	O-Xyl	H	S	102147078

Acteol type							
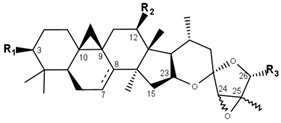
Compounds	R1	R2	R3	Δ^7,8^	24	25	CID
Acteol	OH	OH	OH	-	S	R	59595161
Actein	O-Xyl	O-Ac	OH	-	R	S	10032468
23-*Epi*-26-deoxyactein	O-Xyl	O-Ac	H	-	R	R	10974362
Cimiracemoside N	O-Ara	O-Ac	H	-	S	S	21591918
Cimiracemoside P	O-Xyl	O-Ac	=O	-	S	R	91827183
12-O-Acetylacteol	OH	O-Ac	OH	-	S	S	23640137

Cimiracemoside type			
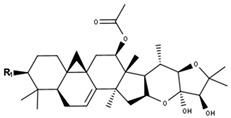
Compounds	R1	Δ^7,8^	CID
Cimiracemoside A (=F)	O-Xyl	+	21606551
Cimiracemoside H	O-Xyl	-	21606553

Neocimigenoside type		
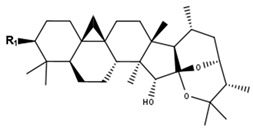
Compounds	R1	CID
Neocimicigenoside A	O-Ara	44583839
Neocimicigenoside B	O-Xyl	44583840

Cimilactone type			
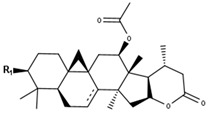
Compounds	R1	Δ^7,8^	CID
Cimilactone A	O-Xyl	-	10908062

Podocarpaside type												
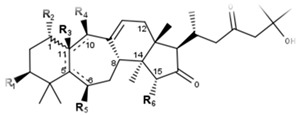
Compounds	R1	R2	R3	R4	R5	R6	Δ^5,6^	Δ^5,11^	Δ^10,11^	5	11	CID
Podocarpaside A	*O*-Ara	OH	-	H	H	H	-	+	-	-	-	16110015
Podocarpaside B	*O*-Ara	H	H	OH	H	H	-	-	-	R	S	16110011
Podocarpaside C	*O*-Ara	H	H	OH	H	OH	-	-	-	R	S	16110016
Podocarpaside D	*O*-Ara	H	OH	H	H	H	-	-	-	S	S	16110012
Podocarpaside E	*O*-Ara	H	-	OH	OH	OH	-	+	-	-	-	139071967
Podocarpaside F	*O*-Ara	H	-	-	H	OH	-	-	+	R	-	16110017
Podocarpaside G	*O*-Ara	H	-	-	-	OH	+	-	+	-	-	16110014

Podocarpaside type												
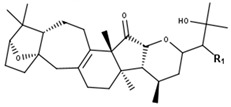
Compounds	R1	R2	R3	R4	R5	R6	Δ^5,6^	Δ^5,11^	Δ^10,11^	5	11	CID
Podocarpaside A	*O*-Ara	OH	-	H	H	H	-	+	-	-	-	16110015
Podocarpaside B	*O*-Ara	H	H	OH	H	H	-	-	-	R	S	16110011
Podocarpaside C	*O*-Ara	H	H	OH	H	OH	-	-	-	R	S	16110016
Podocarpaside D	*O*-Ara	H	OH	H	H	H	-	-	-	S	S	16110012
Podocarpaside E	*O*-Ara	H	-	OH	OH	OH	-	+	-	-	-	139071967
Podocarpaside F	*O*-Ara	H	-	-	H	OH	-	-	+	R	-	16110017
Podocarpaside G	*O*-Ara	H	-	-	-	OH	+	-	+	-	-	16110014

Acerinol type		
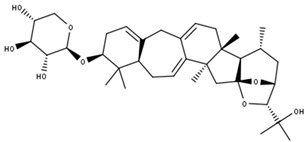
Compounds	R1	CID
24-*O*-Acetylacerionol	O-Ac	101596791
Acerinol	OH	73347277

Cimicinol	
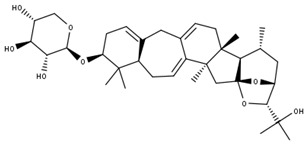
Compound	CID
Cimicinol	102146755

Actaeaepoxide-3-O-beta-D-xylopyranoside	
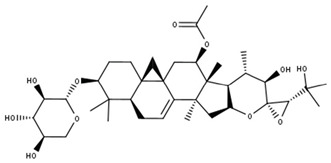
Compound	CID
Actaeaepoxide-3-*O*--*D*-xylopyranoside	15515494

Friedelin	
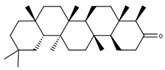
Compound	CID
Friedelin	91472

Cinnamic acid derivatives				
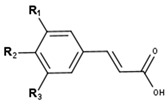
Compounds	R1	R2	R3	CID
Sinapic acid	O-Me	OH	O-Me	637775
*p*-Coumaric acid	H	OH	H	637542
Isoferulic acid	OH	O-Me	H	736186
3,4-Dimethoxycinnamic acid	O-Me	O-Me	H	717531

Cimiracemate type				
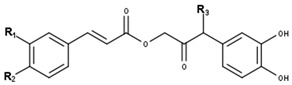
Compounds	R1	R2	R3	CID
Cimiracemate A	OH	O-Me	H	5315874
Cimiracemate B	O-Me	OH	H	5315876
Cimiracemate C	OH	O-Me	O-Me *	5315877
Cimiracemate D	O-Me	OH	O-Me *	5315878
* Stereochemistry not known.				

Cimiciphenone type		
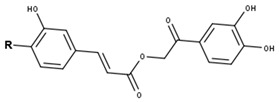
Compounds	R	CID
Cimiciphenone	*O*-Me	71487912
Petasiphenone	OH	16066851

Protocatechuic acid type		
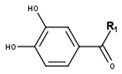
Compounds	R1	CID
Protocatechuic acid	OH	72
Protocatechuic aldehyde	H	637542

Fukiic acid derivatives		
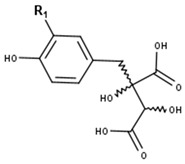
Compounds	R1	CID
Fukiic acid	OH	161871
Piscidic acid	H	120693

Cimicifugic acid derivatives					
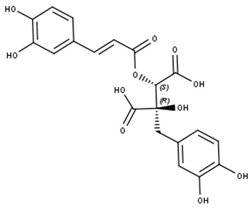
Compounds	R1	R2	R3	R4	CID
Cimicifugic acid A (2-Feruloyl fukinolic acid)	OH	OH	*O*-Me	OH	6449879
Cimicifugic acid B (2-Isoferuloyl piscidic acid)	OH	OH	OH	*O*-Me	6449880
Cimicifugic acid C (2-p Coumaric fukinolic acid)	OH	OH	H	OH	6401178
Cimicifugic acid D (2-Caffeoyl piscidic acid)	OH	H	OH	OH	11742743
Cimicifugic acid E (2-Feruloyl piscidic acid)	OH	H	*O*-Me	OH	10002902
Cimicifugic acid F (2-Isoferuloyl piscidic acid)	OH	H	OH	*O*-Me	6450179
Cimicifugic acid G (2-Feruloyl piscidic acid)	OH	OH	*O*-Me	*O*-Me	11655574
Fukinolic acid	OH	OH	OH	OH	6441059

Actealactone	
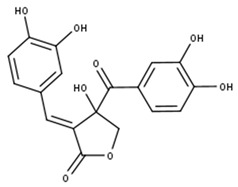
Compound	CID
Actealactone	11537736

Astilbin	
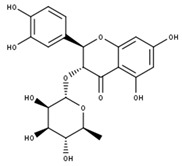
Compound	CID
Astilbin	119258

Chromones						
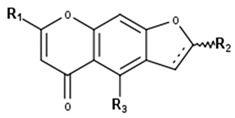
Compound	R1	R2	R3	Δ^7a−7b^	7b	CID
Angelicain	CH_2_-OH	propan-2-ol	OH	-	S	46240156
Cimifugin	*O*-Me	propan-2-ol	*O*-Me	-	S	4411960
Visnagin	Me	H	*O*-Me	+	-	6716

Cimidahurine	
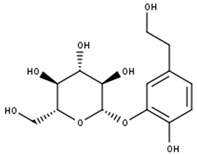
Compound	CID
Cimidahurine	5315870

Cimipronidine		
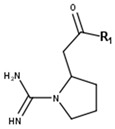
Compounds	R1	CID
Cimipronidine	OH	21594000
Cimipronidine methylester	O-Me	101467166

Dopargine	
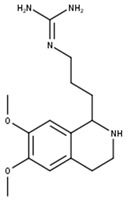
Compound	CID
Dopargine	10357001

Cyclocimipronidine	
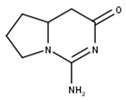
Compound	CID
Cyclocimipronidine	101467165

N-Methycytisine	
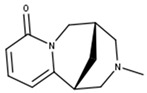
Compound	CID
N-Methycytisine	670971

**Table A2:** Support of classification: similarity of Cimicifuga constituents to database elements.

(Cosine-Similarity Score)											
No	Generic Name	Top 1	Score	Top 2	Sore	Top 3	Score	Top 4	Score	Top 5	Score
Cimi_1	12-beta-Acetoxy-Cimigenol	DMAT	0.902	2-Hydroxyestradiol	0.899	CHEMBL3393133	0.899	CHEMBL3133762	0.899	CHEMBL196759	0.899
Cimi_2	12-beta-Acetyl-Cimigenol-3-O-beta-D-xylopyranoside	CHEMBL3393133	0.951	CHEMBL3133762	0.951	CHEMBL196759	0.951	2-Hydroxyestradiol	0.951	Ezetimibe	0.940
Cimi_3	12-beta-Hydroxy-Cimigenol	DMAT	0.900	CHEMBL196759	0.899	CHEMBL3133762	0.899	2-Hydroxyestradiol	0.899	CHEMBL3393133	0.899
Cimi_4	12-O-Acetylacteol	DMAT	0.903	CHEMBL3133762	0.900	CHEMBL196759	0.900	CHEMBL3393133	0.900	2-Hydroxyestradiol	0.900
Cimi_5	15-O-Methyl-Cimigenol	CHEMBL3393133	0.899	2-Hydroxyestradiol	0.899	CHEMBL3133762	0.899	CHEMBL196759	0.899	CHEMBL3361128	0.887
Cimi_6	23-epi-26-Deoxyactein=27-Deoxyactein	CHEMBL3393133	0.952	CHEMBL196759	0.952	CHEMBL3133762	0.952	2-Hydroxyestradiol	0.952	CHEMBL2325901	0.931
Cimi_7	23-O-Acetylshengmanol	Compound C2	0.909	CHEMBL2017214	0.899	CHEMBL383246	0.895	CHEMBL3963444	0.894	6 Paradol	0.894
Cimi_8	23-O-Acetylshengmanol 3-O-beta-D-xylopyranoside	CHEMBL2325901	0.951	CHEMBL371968	0.934	CHEMBL2420899	0.934	Polydatin	0.934	Teneligliptin	0.934
Cimi_9	23-O-Acetylshengmanol xyloside	CHEMBL2325901	0.951	CHEMBL371968	0.934	CHEMBL2420899	0.934	Polydatin	0.934	Teneligliptin	0.934
Cimi_10	24-Acetylhydroshengmanol xyloside	CHEMBL196759	0.941	CHEMBL3393133	0.941	CHEMBL3133762	0.941	2-Hydroxyestradiol	0.941	CHEMBL2325901	0.939
Cimi_11	24-O-Acetylacerionol	CHEMBL3746293	0.923	GW275944X	0.921	Theasinensis A	0.917	CHEMBL1078665	0.901	Mogrol	0.897
Cimi_12	25-AnhydroCimigenol-3-O-alpha-L-arabinoside	6-O-Cinnamoyl-D-glucopyranose	0.952	GW290597X	0.952	GW458787A	0.952	delphinidin-3-glucoside	0.952	Ezetimibe	0.952
Cimi_13	25-O-AcetylCimigenol	CHEMBL383246	0.901	CHEMBL3963444	0.900	6 Paradol	0.900	CHEMBL4112013	0.900	Ascofuranone	0.878
Cimi_14	25-O-AcetylCimigenol 3-o-alpha-L-arabinoside	CHEMBL3393133	0.951	CHEMBL3133762	0.951	CHEMBL196759	0.951	2-Hydroxyestradiol	0.951	Ezetimibe	0.940
Cimi_15	25-O-Acetyl-cimigenol xyloside	CHEMBL3393133	0.951	CHEMBL3133762	0.951	CHEMBL196759	0.951	2-Hydroxyestradiol	0.951	Ezetimibe	0.940
Cimi_16	25-O-Ethyl-cimigenol-3-O-beta-D-xylopyranoside	2-Hydroxyestradiol	0.951	CHEMBL3393133	0.951	CHEMBL3133762	0.951	CHEMBL196759	0.951	GYY4137	0.928
Cimi_17	25-O-Methyl-cimigenol	CHEMBL3393133	0.899	2-Hydroxyestradiol	0.899	CHEMBL3133762	0.899	CHEMBL196759	0.899	CHEMBL3361128	0.887
Cimi_18	25-O-Methyl-cimigenol-3-O-beta-D-xyloside	2-Hydroxyestradiol	0.951	CHEMBL3393133	0.951	CHEMBL3133762	0.951	CHEMBL196759	0.951	GYY4137	0.928
Cimi_20	3,4-Dimethoxycinnamic acid	4a-Isoalantolactone	0.958	Nootkatone	0.958	Gemcitabine	0.957	Fenoldopam	0.936	GW439255X	0.913
Cimi_22	Acerinol	Theasinensis A	0.917	GW275944X	0.895	Folic caid	0.892	6-O-cinnamoyl-D-glucopyranose	0.890	Karaviloside X	0.886
Cimi_23	Actaeaepoxide 3-O-beta-D-xylopyranoside	GSK978744A	0.945	CHEMBL2338231	0.943	LCZ696	0.943	Tadalafil	0.943	Berteroin	0.943
Cimi_24	Actaealactone	C129	0.859	GW644007X	0.827	Clozapin	0.827	CHEMBL1081678	0.813	CHEMBL4092508	0.812
Cimi_25	Actein	CHEMBL196759	0.941	CHEMBL3133762	0.941	2-Hydroxyestradiol	0.941	CHEMBL3393133	0.941	Ezetimibe	0.931
Cimi_26	Acteol	DMAT	0.902	CHEMBL3133762	0.899	2-Hydroxyestradiol	0.899	CHEMBL3393133	0.899	CHEMBL196759	0.899
Cimi_27	Angelicain	GW644007X	0.899	CHEMBL3730916	0.864	CHEMBL2338229	0.857	CHEMBL204420	0.838	CHEMBL4217199	0.834
Cimi_28	Bugbanoside E	CHEMBL2420899	0.954	Teneligliptin	0.954	Polydatin	0.954	CHEMBL371968	0.954	Cinacalcet	0.954
Cimi_29	Bugbanoside F	Folic acid	0.961	GSK978744A	0.954	GW290597X	0.952	SC4	0.952	6-O-cinnamoyl-D-glucopyranose	0.952
Cimi_30	Caffeic acid	3-O-methylquercetin	1.000	CHEMBL208286	0.977	CHEMBL2408232	0.934	4a-Isoalantolactone	0.914	Nootkatone	0.914
Cimi_31	Caffeic methyl ester	Nootkatone	0.980	4a-Isoalantolactone	0.980	Fenoldopam	0.959	3-O-methylquercetin	0.938	CHEMBL208286	0.917
Cimi_32	Cimicfugoside M	CHEMBL196759	0.961	2-Hydroxyestradiol	0.961	CHEMBL3133762	0.961	CHEMBL3393133	0.961	GYY4137	0.938
Cimi_33	Cimicidanol	Gamma linolenic acid	0.924	Fluvastatin	0.923	CHEMBL1078665	0.913	CHEMBL4114120	0.912	Compound C2	0.912
Cimi_34	Cimicifugic acid C	Glyceolin	0.957	GW780056X	0.926	Oligomycin	0.926	Ibuprofen	0.926	CHEMBL4092508	0.878
Cimi_35	Cimicifugic acid D	Glyceolin	0.957	GW780056X	0.926	Oligomycin	0.926	Ibuprofen	0.926	CHEMBL4092508	0.878
Cimi_36	Cimicifugic acid E	Glyceolin	0.930	GW780056X	0.900	Oligomycin	0.900	Ibuprofen	0.900	Melatonin	0.885
Cimi_37	Cimicifugic acid F	Glyceolin	0.930	GW780056X	0.900	Oligomycin	0.900	Ibuprofen	0.900	Melatonin	0.885
Cimi_38	Cimicifugoside H-1	CHEMBL2420899	0.954	Teneligliptin	0.954	Polydatin	0.954	CHEMBL371968	0.954	Cinacalcet	0.954
Cimi_39	Cimicifugoside H-2	CHEMBL1230171	0.962	Meriolin 1	0.962	CHEMBL2420899	0.953	Polydatin	0.953	Cinacalcet	0.953
Cimi_40	Cimicifugoside H-3	CHEMBL1230171	0.982	Polydatin	0.973	Teneligliptin	0.973	CHEMBL371968	0.973	GW576924A	0.973
Cimi_41	Cimicinol	Folic acid	0.939	GSK978744A	0.934	SC4	0.932	GW290597X	0.932	6-O-cinnamoyl-D-glucopyranose	0.932
Cimi_42	Cimiciphenone	Paroxetine	0.969	CHEMBL4112741	0.921	Melatonin	0.919	CHEMBL4066628	0.904	Ibuprofen	0.904
Cimi_43	Cimidahurine	Gamma-oryzanol	0.973	Atractylenolide III	0.960	Sirtinol	0.937	Zidovudine	0.933	Compound 59	0.926
Cimi_44	Cimifugin	CHEMBL3930006	0.858	GW644007X	0.858	GW708336X	0.852	Palbociclib	0.849	CHEMBL204420	0.844
Cimi_45	Cimigenol	DMAT	0.900	CHEMBL196759	0.899	CHEMBL3133762	0.899	2-Hydroxyestradiol	0.899	CHEMBL3393133	0.899
Cimi_46	Cimigenol xyloside	CHEMBL196759	0.961	2-Hydroxyestradiol	0.961	CHEMBL3133762	0.961	CHEMBL3393133	0.961	GYY4137	0.938
Cimi_47	Cimigol	DMAT	0.900	CHEMBL196759	0.899	CHEMBL3133762	0.899	2-Hydroxyestradiol	0.899	CHEMBL3393133	0.899
Cimi_48	Cimilactone B	GW576924A	0.953	Teneligliptin	0.953	CHEMBL2420899	0.953	polydatin	0.953	Cinacalcet	0.953
Cimi_49	Cimipronidine	CHEMBL1933279	0.778	Cheletyrine	0.764	Pinosylvin	0.758	Nordihydroguaiaretic acid	0.755	CHEMBL3730146	0.723
Cimi_50	Cimipronidine methyl ester	CHEMBL1933279	0.743	Pinosylvin	0.741	GSK182497A	0.713	Cheletyrine	0.710	BDE-209	0.705
Cimi_51	Cimiracemate A	Paroxetine	0.954	Oligomycin	0.922	GW780056X	0.922	Ibuprofen	0.922	CHEMBL4112741	0.907
Cimi_52	Cimiracemate B	Paroxetine	0.954	Oligomycin	0.922	GW780056X	0.922	Ibuprofen	0.922	CHEMBL4112741	0.907
Cimi_53	Cimiracemate C	Paroxetine	0.956	CHEMBL4066628	0.926	Melatonin	0.910	CHEMBL4112741	0.880	CHEMBL4092508	0.878
Cimi_54	Cimiracemate D	Paroxetine	0.956	CHEMBL4066628	0.926	Melatonin	0.910	CHEMBL4112741	0.880	CHEMBL4092508	0.878
Cimi_55	Cimiracemoside A (=F)	GSK978744A	0.963	Ezetimibe	0.962	Monensin	0.952	Folic acid	0.952	GSK192082A	0.945
Cimi_56	Cimiracemoside B	CHEMBL3393133	0.990	CHEMBL3133762	0.990	CHEMBL196759	0.990	2-Hydroxyestradiol	0.990	GYY4137	0.970
Cimi_57	Cimiracemoside C	CHEMBL196759	0.961	2-Hydroxyestradiol	0.961	CHEMBL3133762	0.961	CHEMBL3393133	0.961	GYY4137	0.938
Cimi_58	Cimiracemoside D	CHEMBL3393133	0.951	CHEMBL3133762	0.951	CHEMBL196759	0.951	2-Hydroxyestradiol	0.951	Ezetimibe	0.940
Cimi_59	Cimiracemoside E	CHEMBL2325901	0.981	Cinacalcet	0.962	Teneligliptin	0.962	Polydatin	0.962	CHEMBL371968	0.962
Cimi_60	Cimiracemoside G	GSK978744A	0.963	Ezetimibe	0.962	Monensin	0.952	Folic acid	0.952	GSK192082A	0.945
Cimi_61	Cimiracemoside H	CHEMBL3393133	0.951	CHEMBL3133762	0.951	CHEMBL196759	0.951	2-Hydroxyestradiol	0.951	Ezetimibe	0.940
Cimi_62	Cimiracemoside J	Ezetimibe	0.962	Monensin	0.952	CHEMBL3975011	0.952	CHEMBL4246000	0.952	GSK978744A	0.945
Cimi_63	Cimiracemoside K	Ezetimibe	0.962	Monensin	0.952	CHEMBL3975011	0.952	CHEMBL4246000	0.952	GSK978744A	0.945
Cimi_64	Cimiracemoside L	CHEMBL2325901	0.929	CDN1163	0.918	Cinacalcet	0.914	CHEMBL2420899	0.914	polydatin	0.914
Cimi_65	Cimiracemoside N	CHEMBL3393133	0.952	CHEMBL196759	0.952	CHEMBL3133762	0.952	2-Hydroxyestradiol	0.952	CHEMBL2325901	0.931
Cimi_66	Cimiracemoside P	CHEMBL3133762	0.932	CHEMBL196759	0.932	2-Hydroxyestradiol	0.932	CHEMBL3393133	0.932	CHEMBL2325901	0.931
Cimi_67	Cimiside A	CHEMBL196759	0.961	2-Hydroxyestradiol	0.961	CHEMBL3133762	0.961	CHEMBL3393133	0.961	GYY4137	0.938
Cimi_68	Cimiside B	CHEMBL196759	0.971	2-Hydroxyestradiol	0.971	CHEMBL3133762	0.971	CHEMBL3393133	0.971	GYY4137	0.949
Cimi_69	Cimiside E	6-O-Cinnamoyl-D-glucopyranose	0.952	GW290597X	0.952	GW458787A	0.952	delphinidin-3-glucoside	0.952	Ezetimibe	0.952
Cimi_70	Cyclocmipronidine	Cheletyrine	0.801	15,16-dihydrotanshinone I	0.800	CHEMBL3730933	0.800	Nordihydroguaiaretic acid	0.791	CHEMBL188282	0.757
Cimi_71	Dahurinol	CHEMBL383246	0.901	CHEMBL4094080	0.897	2G11	0.897	Gamma linolenic acid	0.884	CHEMBL3735890	0.884
Cimi_72	Dopargine	Tangeretin	0.768	GSK182497A	0.751	SC-202671	0.748	CHEMBL3927465	0.748	Oleic acid	0.747
Cimi_73	Ferulic acid methyl ester	4a-Isoalantolactone	0.958	Nootkatone	0.958	Gemcitabine	0.957	Fenoldopam	0.936	GW439255X	0.913
Cimi_74	Formononetin	CHEMBL3774632	1.000	SB-409514	0.984	PP487	0.969	CHEMBL3393131	0.969	CHEMBL207674	0.969
Cimi_75	Friedelin	Procyanidin B2	0.891	CHEMBL4112013	0.857	GSK635416A	0.857	CHEMBL3727865	0.850	CHEMBL3859268	0.840
Cimi_76	Fukiic acid	Glyceolin	0.924	Adenine	0.861	CHEMBL2408232	0.861	Icaritin	0.859	Oligomycin	0.853
Cimi_77	Fukinolic acid	Glyceolin	0.957	GW780056X	0.926	Oligomycin	0.926	Ibuprofen	0.926	CHEMBL4092508	0.878
Cimi_78	IsoCimicifugamide	Compound 59	0.869	Nummularic acid	0.858	Sirtinol	0.858	Bupivacaine	0.849	CHEMBL3931350	0.837
Cimi_79	Isoferulic acid	Nootkatone	1.000	4a-Isoalantolactone	1.000	fenoldopam	0.979	3-O-methylquercetin	0.914	Gemcitabine	0.914
Cimi_80	Neocimicigenoside A	CHEMBL196759	0.941	CHEMBL3393133	0.941	CHEMBL3133762	0.941	2-Hydroxyestradiol	0.941	CHEMBL2325901	0.939
Cimi_81	Neocimicigenoside B	CHEMBL196759	0.941	CHEMBL3393133	0.941	CHEMBL3133762	0.941	2-Hydroxyestradiol	0.941	CHEMBL2325901	0.939
Cimi_82	N-Methylcytisine	TBB	0.797	CHEMBL3967075	0.784	Soyasapogenol C	0.780	Momordicoside Q	0.780	GW827396X	0.738
Cimi_83	p-Coumaric acid	Sulforaphane	1.000	3-O-Methylquercetin	0.879	GW782612X	0.868	CHEMBL3736320	0.849	CHEMBL208286	0.847
Cimi_84	Petasiphenone	CHEMBL4112741	0.951	Paroxetine	0.936	Ibuprofen	0.933	GW780056X	0.933	Oligomycin	0.933
Cimi_85	Piscidic acid	Glyceolin	0.892	Adenine	0.891	Icaritin	0.831	Ibuprofen	0.814	Oligomycin	0.814
Cimi_86	Podocarpaside A	CHEMBL1230171	0.962	Meriolin	0.962	CHEMBL2420899	0.953	Polydatin	0.953	Cinacalcet	0.953
Cimi_87	Podocarpaside B	CHEMBL1230171	0.962	Meriolin	0.962	CHEMBL2420899	0.953	Polydatin	0.953	Cinacalcet	0.953
Cimi_88	Podocarpaside C	CHEMBL1230171	0.962	Meriolin	0.962	CHEMBL2420899	0.953	Polydatin	0.953	Cinacalcet	0.953
Cimi_89	Podocarpaside D	CHEMBL1230171	0.962	Meriolin	0.962	CHEMBL2420899	0.953	Polydatin	0.953	Cinacalcet	0.953
Cimi_90	Podocarpaside F	CHEMBL1230171	0.962	Meriolin	0.962	CHEMBL2420899	0.953	Polydatin	0.953	Cinacalcet	0.953
Cimi_91	Podocarpaside G	CHEMBL1230171	0.952	Meriolin	0.952	Teneligliptin	0.944	Polydatin	0.944	CHEMBL371968	0.944
Cimi_92	Protocatechualdehyde	CHEMBL208286	0.951	3-O-Methylquercetin	0.929	Belinostat	0.923	CHEMBL2408232	0.909	GW782612X	0.872
Cimi_93	Protocatechuic acid	CHEMBL208286	1.000	3-O-Methylquercetin	0.977	CHEMBL2408232	0.956	Fenoldopam	0.910	Nootkatone	0.891
Cimi_94	Shengmanol	CHEMBL196759	0.910	DMAT	0.892	CHEMBL3133762	0.889	CHEMBL3393133	0.889	2-Hydroxyestradiol	0.889
Cimi_95	Shengmanol xyloside	CHEMBL196759	0.951	CHEMBL3133762	0.931	2-Hydroxyestradiol	0.931	CHEMBL3393133	0.931	Ezetimibe	0.920
Cimi_96	Sinapic acid	Nootkatone	0.961	4a-Isoalantolactone	0.961	Fenoldopam	0.941	Gemcitabine	0.920	CHEMBL4066628	0.895
Cimi_97	Visnagin	Prednisolone	0.938	CHEMBL3976646	0.889	Rifampicin	0.889	CHEMBL208118	0.889	Monascus	0.889
Cimi_2_ metab	12-beta-Acetoxycimigenol	DMAT	0.902	2-Hydroxyestradiol	0.899	CHEMBL3393133	0.899	CHEMBL3133762	0.899	CHEMBL196759	0.899
Cimi_6_ metab	Cimi_6_metab	CHEMBL3393133	0.910	CHEMBL196759	0.910	2-Hydroxyestradiol	0.910	CHEMBL3133762	0.910	Monensin	0.908
Cimi_8_ metab	23-O-Acetylshengmanol	Compound C2	0.909	CHEMBL2017214	0.899	CHEMBL383246	0.895	CHEMBL3963444	0.894	6 Paradol	0.894
Cimi_9_ metab	Cimi_9_metab	Compound C2	0.909	CHEMBL2017214	0.899	CHEMBL383246	0.895	CHEMBL3963444	0.894	6 Paradol	0.894
Cimi_10_ metab	Cimi_10_metab	CHEMBL196759	0.899	2-Hydroxyestradiol	0.899	CHEMBL3133762	0.899	CHEMBL3393133	0.899	CHEMBL4094080	0.899
Cimi_12_ metab	Cimi_12_metab	Ascofuranone	0.919	AKOS007865932	0.907	CHEMBL4246000	0.907	CHEMBL3975011	0.907	delphinidin-3-glucoside	0.898
Cimi_14_ metab	Cimi_14_metab	DMAT	0.902	2-Hydroxyestradiol	0.899	CHEMBL3393133	0.899	CHEMBL3133762	0.899	CHEMBL196759	0.899
Cimi_15_ metab	Cimi_15_metab	DMAT	0.902	2-Hydroxyestradiol	0.899	CHEMBL3393133	0.899	CHEMBL3133762	0.899	CHEMBL196759	0.899
Cimi_16_ metab	25- O-Methylcimigenol	CHEMBL3393133	0.899	2-Hydroxyestradiol	0.899	CHEMBL3133762	0.899	CHEMBL196759	0.899	CHEMBL3361128	0.887
Cimi_18_ metab	25-O-Methylcimigenol	CHEMBL3393133	0.899	2-Hydroxyestradiol	0.899	CHEMBL3133762	0.899	CHEMBL196759	0.899	CHEMBL3361128	0.887
Cimi_19_ metab	Cimi_19_metab	DMAT	0.903	CHEMBL3133762	0.900	CHEMBL196759	0.900	CHEMBL3393133	0.900	2-Hydroxyestradiol	0.900
Cimi_22_ metab	Cimi_22_metab	Monensin	0.909	GSK978744A	0.905	CHEMBL2338231	0.903	Berteroin	0.903	LCZ696	0.903
Cimi_23_ metab	Cimi_23_metab	Monensin	0.909	GSK978744A	0.905	CHEMBL2338231	0.903	Berteroin	0.903	LCZ696	0.903
Cimi_25_ metab	Cimi_25_metab	DMAT	0.903	CHEMBL3133762	0.900	CHEMBL196759	0.900	CHEMBL3393133	0.900	2-Hydroxyestradiol	0.900
Cimi_28_ metab	Cimi_28_metab	CHEMBL3746293	0.925	CHEMBL1078665	0.925	Compound C2	0.924	CHEMBL4114120	0.902	GW275944X	0.902
Cimi_29_ metab	Cimi_29_metab	CHEMBL2376144	0.899	CHEMBL2041962	0.898	CHEMBL3427184	0.898	Epiberberine	0.898	GW275944X	0.898
Cimi_32_ metab	Cimi_32_metab	DMAT	0.900	CHEMBL196759	0.899	CHEMBL3133762	0.899	2-Hydroxyestradiol	0.899	CHEMBL3393133	0.899
Cimi_38_ metab	Cimicidanol	Gamma linolenic acid	0.924	Fluvastatin	0.923	CHEMBL1078665	0.913	CHEMBL4114120	0.912	Compound C2	0.912
Cimi_39_ metab	Cimi_39_metab	Gamma linolenic acid	0.966	Fluvastatin	0.966	CHEMBL2337767	0.955	Epiberberine	0.955	CHEMBL1078665	0.933
Cimi_40_ metab	Cimi_40_metab	C128	0.956	Pitavastatin	0.955	Xanthohumol	0.928	Fluvastatin	0.920	CHEMBL4114120	0.909
Cimi_41_ metab	Cimi_41_metab	GW631581B	0.894	Theasinensis A	0.884	Compound C2	0.884	GW275944X	0.881	Folic acid	0.880
Cimi_43_ metab	Hydroxytyrosol	CHEMBL1233881	1.000	CHEMBL4112741	0.830	CHEMBL3909286	0.823	gamma-oryzanol	0.802	CHEMBL4215572	0.793
Cimi_46_ metab	Cimigenol	DMAT	0.900	CHEMBL196759	0.899	CHEMBL3133762	0.899	2-Hydroxyestradiol	0.899	CHEMBL3393133	0.899
Cimi_56_ metab	Cimi_56_metab	CHEMBL3393133	0.941	2-Hydroxyestradiol	0.941	CHEMBL3133762	0.941	CHEMBL196759	0.941	CHEMBL4278763	0.917
Cimi_57_ metab	Cimigenol	DMAT	0.900	CHEMBL196759	0.899	CHEMBL3133762	0.899	2-Hydroxyestradiol	0.899	CHEMBL3393133	0.899
Cimi_58_ metab	12beta-acetoxycimigenol	DMAT	0.902	2-Hydroxyestradiol	0.899	CHEMBL3393133	0.899	CHEMBL3133762	0.899	CHEMBL196759	0.899
Cimi_59_ metab	Cimi_59_metab	CHEMBL2325901	0.939	CHEMBL2420899	0.923	CHEMBL371968	0.923	Teneligliptin	0.923	Polydatin	0.923
Cimi_61_ metab	Cimi_61_metab	CHEMBL3393133	0.920	CHEMBL3133762	0.920	CHEMBL196759	0.920	2-Hydroxyestradiol	0.920	Monensin	0.917
Cimi_62_ metab	Cimi_62_metab	Ascofuranone	0.923	AKOS007865932	0.911	Monensin	0.908	CHEMBL3975011	0.908	CHEMBL4246000	0.908
Cimi_63_ metab	Cimi_63_metab	Ascofuranone	0.923	AKOS007865932	0.911	Monensin	0.908	CHEMBL3975011	0.908	CHEMBL4246000	0.908
Cimi_64_ metab	23-O-Acetylshengmanol	Compound C2	0.909	CHEMBL2017214	0.899	CHEMBL383246	0.895	CHEMBL3963444	0.894	6 Paradol	0.894
Cimi_65_ metab	Cimi_65_metab	CHEMBL3393133	0.910	CHEMBL196759	0.910	2-Hydroxyestradiol	0.910	CHEMBL3133762	0.910	Monensin	0.908
Cimi_66_ metab	Cimi_66_metab	CHEMBL2017214	0.911	DMAT	0.903	CHEMBL3735890	0.889	CHEMBL383246	0.885	CHEMBL3393133	0.879
Cimi_67_ metab	12beta-Hydroxycimigenol	DMAT	0.900	CHEMBL196759	0.899	CHEMBL3133762	0.899	2-Hydroxyestradiol	0.899	CHEMBL3393133	0.899
Cimi_68_ metab	Cimigenol	DMAT	0.900	CHEMBL196759	0.899	CHEMBL3133762	0.899	2-Hydroxyestradiol	0.899	CHEMBL3393133	0.899
Cimi_69_ metab	Cimi_69_metab	Ascofuranone	0.919	AKOS007865932	0.907	CHEMBL4246000	0.907	CHEMBL3975011	0.907	delphinidin-3-glucoside	0.898
Cimi_78_ metab	Cimi_78_metab	SB-732941	0.924	CHEMBL3933251	0.875	CHEMBL3728128	0.870	Corosolic acid	0.852	Hernandezine	0.800
Cimi_80_ metab	Cimi_80_metab	Urolithin A	0.907	CHEMBL2017214	0.897	CHEMBL383246	0.891	DMAT	0.890	CHEMBL3963444	0.890
Cimi_81_ metab	Cimi_81_metab	Urolithin A	0.907	CHEMBL2017214	0.897	CHEMBL383246	0.891	DMAT	0.890	CHEMBL3963444	0.890
Cimi_86_ metab	Cimi_86_metab	Gamma linolenic acid	0.955	CHEMBL1078665	0.944	CHEMBL4114120	0.943	GW780159X	0.942	Crocin	0.941
Cimi_87_ metab	Cimi_87_metab	CHEMBL4114120	0.953	Crocin	0.952	Gamma linolenic acid	0.943	Fluvastatin	0.941	Fucoxanthin	0.940
Cimi_88_ metab	Cimi_88_metab	Gamma linolenic acid	0.955	Fluvastatin	0.954	CHEMBL1078665	0.944	CHEMBL4114120	0.943	Epiberberine	0.942
Cimi_89_ metab	Cimi_89_metab	CHEMBL4114120	0.953	Crocin	0.952	Gamma linolenic acid	0.943	Fluvastatin	0.941	Fucoxanthin	0.940
Cimi_90_ metab	Cimi_90_metab	CHEMBL1230171	0.962	Meriolin 1	0.962	CHEMBL2420899	0.953	polydatin	0.953	Cinacalcet	0.953
Cimi_91_ metab	Cimi_91_metab	Gamma linolenic acid	0.943	Fluvastatin	0.941	CHEMBL1078665	0.932	CHEMBL4114120	0.930	Epiberberine	0.929
Cimi_92_ metab	Cimi_92_metab	CHEMBL196759	0.910	DMAT	0.892	CHEMBL3133762	0.889	CHEMBL3393133	0.889	2-Hydroxyestradiol	0.889
Median			0.941		0.923		0.922		0.912		0.907

Highlighted in blue are the constituents for which no comparison molecule was found in the database with a cosine similarity score > 0.8.
